# Verbal fluency and semantic association deficits in children with in birth nonprogressive neuromuscular diseases

**DOI:** 10.3389/fnhum.2025.1499521

**Published:** 2025-02-06

**Authors:** Maria Koriakina, Olga E. Agranovich, Ioannis Ntoumanis, Maxim Ulanov, Isak B. Blank, Anna Shestakova, Evgeny Blagovechtchenski

**Affiliations:** ^1^Affective Psychophysiology Laboratory, Institute of Health Psychology, HSE University, Moscow, Russia; ^2^Centre for Cognition and Decision Making, Institute for Cognitive Neuroscience, HSE University, Moscow, Russia; ^3^H.Turner National Medical Research Center for Children’s Orthopedics and Trauma Surgery of the Ministry of Health of the Russian Federation, St. Petersburg, Russia

**Keywords:** motor skills, cognitive skills, motor disorders, semantic association, verbal fluency, embodiment cognition

## Abstract

**Introduction:**

The relationship between motor and cognitive skills is a pivotal issue in neuroscience, with embodied cognition theory asserting that bodily actions and experiences play a vital role in cognitive processing. This relevance is particularly noted in children with severe motor disorders (MD) from birth, highlighting a need to explore how these disorders may impede cognitive functions.

**Methods:**

In this study, we assessed verbal fluency, a critical component of speech function, in 68 children aged 7 to 15. This group consisted of 36 children with motor disorders, specifically those diagnosed with obstetric brachial plexus palsy (OBPP, *n* = 22) or arthrogryposis multiplex congenita (AMC, *n* = 14), and 32 healthy control children. We compared levels of verbal fluency, action/verbal naming, and the development of semantic associations between the two groups.

**Results:**

The findings revealed that children with motor impairments exhibited significantly lower performance in tasks measuring verbal fluency and semantic association compared to the control group. Mainly, MD children produced fewer words during verbal fluency tasks and demonstrated reduced semantic associations. Interestingly, MD children with unilateral limb impairment outperformed those with bilateral impairment on semantic association tasks.

**Discussion:**

These results suggest that the cognitive deficits observed in children with motor impairments can be attributed to less engagement with their physical environment, which influences their ability to perceive and manipulate objects differently based on their level of impairment. Additionally, the findings underscore how social and cultural contexts may be affected by motor impairments. Overall, our study supports the concept of embodied cognition, demonstrating that delays in motor skill development among children with OBPP and AMC can harm their cognitive functions.

## 1 Introduction

Numerous studies have shown different types of relationships between cognitive and motor development (Houwen et al., 2016). The question of how strongly these functions are related remains open, and several interpretations of this phenomenon exist in the literature. In this study, we attempted to assess how a sensitive cognitive function like speech—particularly verbal fluency and semantic associations—is associated with deficits in motor development from birth.

Previous studies have shown that children’s active development of fine motor skills promotes effective language skills development (Bedford et al., 2016). These studies also suggest that good hand-related motor skills are associated with improved verbal fluency (Beeks, 2013). Brain activity related to motor skills correlates with increased connectivity between the brain areas responsible for language functions (Dayan and Cohen, 2011). The connection between motor skills and speech development can also be explained by the close connection between motor and language processing in the brain (Hillman et al., 2020).

The engagement in body movements activates specific areas of the brain, including the motor areas of the cerebral hemispheres (Willems et al., 2009). These areas are in close contact with the language-processing areas of the brain (Friederici, 2011). In addition, improving motor skills can lead to better coordination of tongue movements, which can also contribute to more effective use of speech mechanisms (Stipancic et al., 2021). Further research is necessary to gain a more detailed and accurate understanding of the relationship between motor skills and speech development.

One explanation for explanation for the interaction between motor and cognitive skills is offered by the theory of embodied cognition, which suggests that our physical abilities influence how we perceive and estimate the world, ultimately shaping our experiences (Foglia and Wilson, 2013). Embodiment simulation is another term that has been introduced to describe the mechanism by which the interplay between the brain and body emerges, positing that cognitive processes are deeply intertwined with the physical body and its interactions with the environment (Gallese and Lakoff, 2005).

It is thought that a child’s overall activity—particularly the development achieved through movement and the acquisition of new motor skills—initiates a cascade of changes that extend beyond motor production (Hestbaek et al., 2017). These changes affect various domains, including perception and cognition, language and communication, emotional expression and regulation, and physical growth and health. In light of this, it seems likely that children with congenital motor disorders have different speech development from healthy children from birth.

The purpose of this study was to assess the level of speech development in children with in birth motor impairments and compare them to healthy children. Some studies have shown that there are specific features in the relationship between motor and speech functions in children with motor disorders (Iverson, 2010) and that motor dysfunction is often associated with speech disorders (Visscher et al., 2007); specifically, 70% of children with speech delays have motor problems (Guerra and Cacabelos, 2019). Delayed development of fine motor skills in a child can lead to persistent speech disorders in the future (Bal et al., 2020).

These motor impairments can impact a child’s ability to produce clear speech and can affect their overall communication abilities, highlighting the complex link between motor skills and speech development, especially the specifics of this relationship in children with motor impairments. Previous study shows that early and systematic speech therapy, which considers a child’s motor features, can significantly improve their speech skills and overall quality of life (Pennington et al., 2004). It is important to understand that each child with a motor disorder has unique needs and requires an individual approach to therapy for speech and motor impairments.

Studies of patients with motor disorders have indicated that impaired motor function affects speech function, but the impact of motor disorders on speech in children has not been sufficiently studied. The relationship between motor and cognitive functions requires further empirical attention, particularly the impact of diseases such as arthrogryposis multiplex congenita (ÀMC) and obstetric brachial plexus palsy (OBPP). These are congenital conditions that fundamentally alter a child’s perception of the world from a very young age. Their motor issues are static, meaning they do not worsen over time, but remain consistent, which allows for a predictable framework within which therapies and interventions can be implemented.

AMC is one of the most serious congenital malformations and is characterized by the presence of two or more major joint contractures, muscle aplasia or hypoplasia, and motoneuronal dysfunction in the anterior horns of the spinal cord. The lack of active movement in the joints of the upper extremities is one of the main problems causing limitations or an inability to self-care. OBPP is an injury to the brachial plexus that occurs during birth, usually as a result of a stretching injury from a difficult vaginal delivery. This results in paralysis of the upper limbs.

Children with AMC and OBPP face substantial challenges not only with physical movements but also in their speech development. The precise coordination required for speech production can be hindered by motor impairments, potentially leading to delays and difficulties in articulation, phonation, and overall speech fluency. Moreover, motor impairments can influence the development of cognitive skills, as physical interaction with the environment is crucial for learning and cognitive development during early childhood. This interaction enables exploration, experimentation, and the development of problem-solving skills. When motor abilities are compromised, these opportunities are often limited, which can have cascading effects on cognitive development. Thus, we predicted that the lack of motor activity in these children’s limbs would affect their speech abilities.

In this study, speech abilities were examined using a set of tests assessing three speech parameters: verbal fluency (VF), semantic associations (SA), and naming production (NP). Each test focused on different functions related to different brain activities (ref). Speech tests are an important indicator of cognitive processes, especially executive functions. Fluency tests can also demonstrate the effect of motor disease on speech and cognitive function.

Previous studies have shown that children with motor impairments demonstrate deficits in memory and attention compared to healthy children (Koriakina et al., 2021). Children with AMC and OBPP differ in terms of their motor functionality assessment, but cognitive development in both groups is lower than in healthy children (Blagovechtchenski et al., 2023). The absence of motor activity itself may be important in this development.

In this context, the theory of embodied cognition suggests that modality-specific systems for perception, action, and emotion play a crucial role in language processing (Zwaan, 2003). Embodiment cognition theory suggests that the brain constructs representations of actions, sensations, and emotions by simulating these experiences (Harbourne and Berger, 2019). These simulations are related to the sensorimotor systems of the body. For instance, when subjects perceive someone else acting, their motor systems are activated as if they were acting themselves. This process is sometimes associated with the idea of facilitating the understanding and prediction of others’ actions, which is important for social interaction and learning. To effectively simulate these aspects, one needs to have actually had those experiences (in the “here and now”), especially during acquisition, to become part of the semantic representations (Reggin et al., 2023).

While much of the empirical support for this view has focused on adult cognition (Horchak et al., 2014), increasingly extensive research indicates that children also use this sensorimotor simulation to learn vocabulary and develop conceptual knowledge (Wellsby and Pexman, 2014). For children, the capacity to perform sensorimotor simulations arises from the interaction between their developing sensorimotor and affective systems and their experiences with the external world. This includes the physical environment (objects that can be perceived, and manipulated, and that often evoke specific emotions), and the cultural contexts in which the child is immersed. However, children born with these motor disorders, cannot manipulate objects and repeat actions, which likely affects their ability to utilize sensorimotor simulation.

We hypothesized that patients with in birth nonprogressive motor impairments such as AMC and OBPP would have lower scores in verbal fluency, semantic association, and production naming than healthy children. Our objective was to determine whether there was a noticeable difference in speech patterns between children with motor impairments and healthy children. Since speech is a highly sensitive indicator of various developmental processes, it can provide valuable insights into the broader impact of these motor challenges on overall cognitive and linguistic development.

## 2 Materials and methods

### 2.1 Participants

A group of 36 children aged 7–15 years (mean = 10.3) with motor disorders (22 subjects with AMC and 14 subjects with OBPP) was selected from the Turner National Medical Research Center for Children’s Orthopedics and Trauma Surgery. A group of 32 healthy children (15 girls) aged 7–15 years (mean = 9.6) with no history of visual, hearing, or cognitive disorders was selected as a control group. The ratio of girls to boys is about the same in all groups—we have reflected this in Supplementary Table S1. The control group was selected based on the age range of the children diagnosed. To reduce the age spread, the ages of both groups were predominantly the same.

Children diagnosed with AMC and OBPP exhibit several shared pathological traits according to orthopedic classifications. They are considered birth nonprogressive neuromuscular diseases and include the presence of contractures in two or more major joints, hypoplasia or aplasia of muscle tissue, and indications of motoneuron dysfunction in the anterior horn of the spinal cord. The limb presentations of affected individuals manifest a specific profile consisting of the following features: an adductor contracture at the shoulder/knee joint, an extensor contracture in the elbow (with flexion contractures occurring less frequently), a flexion contracture at the wrist, flexion contractures in the fingers, an abduction contracture of the thumb, and either hypoplasia or absence of the limb muscles. Additionally, there is often significant restriction or a complete lack of ability to perform self-care activities. Consequently, we included both AMC and OBPP in our study as part of the same clinical group. Children were evaluated by specialists in other areas and had no cognitive, mental, or other pathological changes.

Since the number of patients with these diseases is limited, children in the healthy group were age-matched to the patient group to maintain sample consistency. Moreover, in our initial studies, we examined a group of children with similar muscular disorders and took their ages into account. Research shows that age does not noticeably affect speech development in these children (Koriakina et al., 2021; Ntoumanis et al., 2021).

### 2.2 Speech tasks

In this study, three speech parameters were assessed: verbal fluency, semantic associations, and naming production. VF tasks allow for the assessment of both the integrity of the lexico-semantic memory and the ability to retrieve items from it to produce verbal material (Patterson and Hayes, 1977). They reflect executive control and memory functions (Kavé and Sapir-Yogev, 2020; Whiteside et al., 2020). NP tasks are widely used to assess oral production disorders. The SA task consists of matching two drawings that have a semantic relationship with each other; this type of task is associated with semantic memory, working memory, and procedural memory (Matthews, 2015).

A set of pictures was selected from different databases such as Snodgrass and Vanderwart, Object and Action naming Battery (Masterson, 2000), or IPNP database (Szekely et al., 2004). The order of the tasks for all subjects was the same, based on the image numbers from the provided database.

### 2.3 Verbal fluency tasks

In verbal fluency tasks, participants are given one minute to produce as many unique words as possible within a specific semantic category (category fluency) or starting with a given letter (letter fluency). The participant’s score in each task is the number of unique correct words. For each verbal fluency task, the participants were asked to generate as many words as possible within 60 s, avoiding repetitions and proper names. All participant utterances were recorded to be coded later. These tasks evaluate the ability to access and retrieve linguistic information from a semantic system (lexical and semantic access, selection of phonemes, and selection of motor patterns), as well as its modulation or alteration depending on the specific modality and thus the neural network in the somatosensory system associated with its processing (i.e., frontal for actions, posterior for objects/animals). The verbal fluency task is unified and not related to the child’s age. Only the number of correct words is assessed when presented with a particular picture.

#### 2.3.1 Automatic naming

Participants were asked to name months of the year.

#### 2.3.2 Animals

Participants were given 60 s to produce as many words as possible that belonged to the category of animals (with an example provided, e.g., lion). They were asked to avoid repeated words and proper names or places.

#### 2.3.3 Musical instruments

The instructions were the same as the animal’s task.

#### 2.3.4 Occupation

The instructions were the same as the animal’s task.

#### 2.3.5 Actions

Participants were given 60 s to produce as many words as possible that belonged to the category of actions, things that could be done by themselves or someone else (with an example provided, e.g., sleep). They were asked to avoid repeated words and proper names or places.

#### 2.3.6 Letter fluency

Participants were given 60 s to produce as many words as possible that start with the letter T (with an example provided, e.g., tiger). They were asked to avoid repeated words and proper names or places.

### 2.4 Naming production tasks

#### 2.4.1 Object picture naming

**FIGURE 1:**
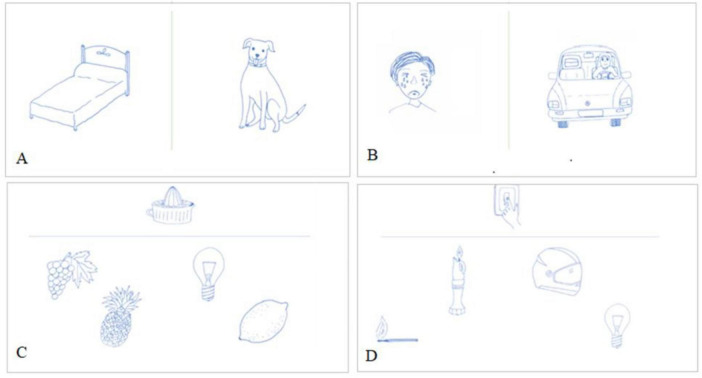
Examples of assignments (**A** - «Object picture naming»; **B** - «Action picture naming»; **C** - «Object-object pairing»; **D** - «Object-action pairing»).

It consists of 30 drawings, half of which correspond to living beings and the other half to inanimate objects (Figure 1A). When the patient has problems at the semantic level (difficulty in generating words or phrases that accurately convey a particular meaning, word-finding difficulties, inability to find the correct word, resulting in pauses or substitutions with semantically similar but incorrect terms), he will have more difficulties with a specific category (living beings or inanimate objects). Instructions: The participant was shown every picture and tasked with identifying the corresponding drawing.

#### 2.4.2 Action picture naming

Thirty drawings of joint actions (half object—actions, the other half is action—object) (Figure 1B). It means that you need to choose the right picture of an object, which compares with action and vise-versa. Instructions: The participant was presented with each illustration and asked about the character’s actions. They were also asked about the protagonist’s activities and what the character was doing.

### 2.5 Semantic association tasks

#### 2.5.1 Object–object pairing

This task consists of 30 items, half of which correspond to living beings and half to inanimate beings (Figure 1C). On each page, there are five drawings, and the patient must decide which of the four drawings on the bottom of the page is related to the drawing on the upper part. The distractors are two semantics and one unrelated.

#### 2.5.2 Object–action and action-object pairing

This task aims to study selective disorders in processing objects\action or actions\object. It consists of 30 plates with five drawings each (an action and four objects or an object and four actions) (Figure 1D). In the first half, the patient must connect the action with one of the four objects presented; in the other half, an object with one of the four actions presented.

To compare the speech fluency of groups of patients, we separated them into two groups: those with one affected limb and those with more than one affected limb (for example, with two affected hands and one affected leg). This separation allowed us to test our hypothesis about the relationship between motor activity and speech function. Children with only one affected hand have a greater range of motor activity and a greater ability to interact with objects than children with two or more affected limbs.

## 3 Statistical analysis

For every category of the speech ability tasks, we conducted a Mann–Whitney U test to compare the scores between patients and healthy controls and between the different patient groups (i.e., AMC and OBPP). We selected a non-parametric test because of the non-normal distribution of the data, as assessed by a Shapiro–Wilk normality test (*p* < 0.05). We repeated the same analysis for the additional fluency variables, such as the action picture naming task.

A correction for multiple comparisons using the Holm–Bonferroni method with a significance level of 0.05 was applied to account for the probability of a false positive result when performing multiple comparative tests. The significance threshold was adjusted based on the number of comparative tests conducted. All results and conclusions were considered with this correction adjustment for multiple comparisons.

## 4 Results

**FIGURE 2:**
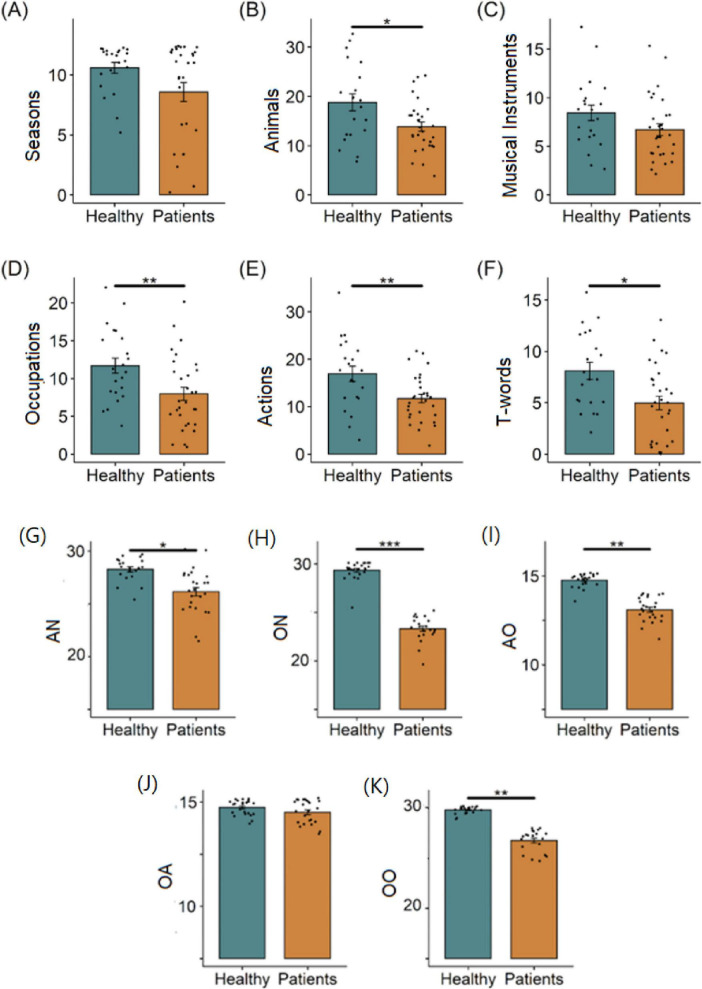
Comparison of healthy children and patients (AMC or OBPP) by the number of correct words named in each task. Verbal fluency tasks: **(A)** seasons, **(B)** animals, **(C)** musical instruments, **(D)** occupations, **(E)** actions, **(F)** words beginning with the letter T. Naming production tasks: **(G)** AN – action naming, **(H)** ON – object naming. Semantic association tasks: **(I)** AO – action–object association, **(J)** OA – object–action association, **(K)** OO – object–object association. The Mann–Whitney U criterion was used for statistical comparisons. *p < 0.05, **p < 0.01, ***p < 0.001.

First, we analyzed all speech tests to determine the differences between the two groups of children with different diagnoses. The results for children in the motor development disorder group did not differ depending on whether they were diagnosed with AMC or OBPP. The next step of the analyses involved comparing the patient group (all children with AMC or OBPP) with the control group of typically developing children across all speech assessments (see Figure 2).

As shown in Figure 2, the healthy children had a significantly higher VF index than those with motor disorders. This indicates significant deviations from the norm in the structure of cognitive processes or the patients’ word retrieval strategies. The healthy children also had a significantly higher semantic association index than those with motor disorders in the action–object and object–object association tasks and a considerably higher naming production index than those with motor disorders. This indicates that children with motor disorders have significant deviations from the norm in the structure of their semantic memory, procedural memory, and working memory.

**FIGURE 3:**
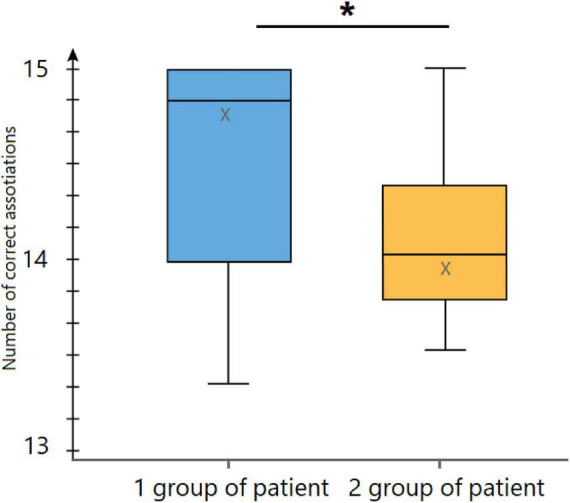
Children diagnosed with AMC and OBPP exhibit a range of disease patterns, each with a unique impact on their daily lives. Whether it’s the loss of function in one arm or the more complex challenges of two arms and one leg, these variations significantly alter the child’s ability to interact with the world and objects. Hence, we have categorized the children into two groups based on the number of affected limbs: (1) patients with one affected hand and (2) patients with more than one affected limb, and compared them on speech tests. We found the differences between the two groups in the Semantic action–object association test. Mann–Whitney U tests were used for statistical comparisons. *p < 0.05. A Mann–Whitney U comparison of the two groups of patients showed that the groups differed on the index of action–object association. Children with more than one affected limb performed worse on this test than children with one affected limb (see Figure 3).

We then compared the two groups of patients (see Figure 3) according to the number of upper limbs affected by AMC or OBPP. The first group included children with one affected hand (10 patients), and the second group had more than one affected limb (13 patients). It is crucial to emphasize that not all children were assessed for affected limbs. However, we identified two groups among those affected: those with a single affected limb and those with multiple ones. This approach allowed us to form two additional samples for a more detailed analysis of this factor’s influence on the study’s results.

## 5 Discussion

The results of the present study showed that children with birth nonprogressive neuromuscular diseases (AMC or OBPP) had worse results on verbal fluency tests than healthy children. Differences were observed in naming animals, professions, actions, and words beginning with the letter T. The VF test requires executive control of cognitive processes such as selective attention, selective inhibition, mental attitude change, internal response formation, self-control, and the ability to retrieve certain information within limited search parameters (Lezak et al., 2012). Notably, beyond language, speech fluency also reflects executive control and memory functions (Kavé and Sapir-Yogev, 2020; Whiteside et al., 2020). This is probably because lexico-semantic memory and the ability to extract elements from it to create verbal material is worse in children with motor disorders than in healthy controls. This also suggests that children with motor disorders have reduced executive control and memory functions.

The findings showed that the children with motor impairments exhibited inferior performance in semantic association assessments, particularly in tasks where it was necessary to associate an object with another object and an action with an object. The SA task consists of matching two drawings that have a semantic relationship with each other. Subjects with a problem at this level (semantic agnosia) are unable to easily relate concepts according to their meaning.

This type of task is associated with semantic memory, working memory, and procedural memory (Matthews, 2015). Semantic memory refers to acquired knowledge about the world, and the structures of the anterior and inferior temporal lobes are most often associated with semantic memory loss (Salmon et al., 1999). This may indicate that children with motor impairments lack acquired knowledge about the world, especially about interaction with objects, because of a deficit in movement. They may also have problems with the structures of the anterior and lower temporal lobes, which are most often associated with the loss of semantic memory. Moreover, children with limb impairment may experience difficulties in associating actions with objects due to their lack of interaction with objects. They may also struggle to utilize sensorimotor effects to learn new vocabulary and understand object functions and actions.

The children with motor disorders also had worse results than the control group on tests for naming objects and actions depicted in pictures. This confirms that children with motor disorders have difficulty accessing the meanings of objects and actions while remembering the picture’s names, and have problems with semantic memory. Naming production is a complex neural process. It involves converting an idea that the speaker wants to convey into a linguistic structure and then translating this linguistic structure into a quick, precisely coordinated articulatory motor sequence (Liljeström et al., 2015).

These results are interesting to consider in the context of the theory of embodied cognition. Other studies have also shown that children born with motor disorders often face significant challenges in speech and language development (Banu et al., 2024). Children with motor impairments may have difficulties pronouncing sounds, creating words and phrases, understanding speech, and interpreting the meaning of words (And and Dodd, 1996).

Nevertheless, this viewpoint is not unanimous, and some studies have reported contradictory findings. Some recent reviews on this topic report no significant correlation between motor and cognitive skills in 4–6-year-old healthy children (van der Fels et al., 2015). Some studies indicate that motor development can significantly aid children with cognitive delays, whereas children with typical cognitive development may not experience similar advancements in these functions (Shi and Feng, 2022). This suggests that children with cognitive delays have more potential for cognitive improvement through targeted motor skills interventions. Cognitive development encompasses a broad array of domains, and the development of motor skills significantly impacts cognitive growth (Shi and Feng, 2022). However, additional research is warranted to ascertain which cognitive markers undergo the most effective development during motor activities.

It is also interesting to consider the obtained results in light of the discussion of the motor system’s role in the functioning of the so-called dorsal and ventral pathways in developing human speech functions. Some views propose a dorsal motor versus ventral semantic division (Ries et al., 2019). Of course, one would like to refer to the system responsible for the so-called “how” program, i.e., the dorsal system (Saur et al., 2008). However, we believe such a direct reference is impossible without functional brain mapping in patients with motor disorders. In this study, we see only the manifestation of disorders at the spinal cord level in features associated with verbal fluency. MRI studies in such children show the primary deviations in healthy children only in the amygdala area (Kadieva et al., 2024).

In the present study, we observed that children with limb motor disease performed significantly worse on speech abilities than children in the control group. This suggests difficulties in the cognitive development of children with motor disease because the problems of speech function could be only the manifestation of general problems with other cognitive functions (Koriakina et al., 2021; Blagovechtchenski et al., 2023) Furthermore, a comparative analysis between groups of children with movement disorders in one hand vs. more than one limb showed that children with only one affected hand handled the “action-object” semantic association task better than children with two affected hands. Discussions about correlations between motor disorders and cognitive impacts remain very important (Blagovechtchenski et al., 2023).

Our findings may be attributable to diminished motor activity, which reflects a decreased capacity for the independent manipulation of objects within the patient’s daily environment. As we engage with the world, we form semantic associations through interactions with surrounding objects (Weidemann et al., 2019). The lack of such interaction could reduce their capacity to explore and identify associative connections. Speech ability tests alone may not be sufficient indicators of cognitive underdevelopment due to motor disease. Nevertheless, the growing interest in using these tests is due to their sensitivity to dysfunction in many conditions associated with functional and structural disorders and to specific cognitive processes (Ntoumanis et al., 2021; Bredikhin et al., 2023).

In addition, some studies show a connection between the level of verbal fluency and executive functioning (EF) (Gustavson et al., 2019). In neuropsychology, executive functions refer to a system of high-level processes that allows one to plan current actions according to a common goal, change responses according to the context, and selectively pay attention to the right stimuli. Across all analyses, verbal fluency had stronger relationships with EF factors, indicating a large executive component involved in the task. There may be a general problem with speech abilities on all assignments. Both phonemic and semantic fluency were equally related to several dimensions of EF and word knowledge and can be viewed as executive language tasks (Aita et al., 2019).

Commands of voluntary movements may be formed with the aid of speech. Preliminary planning, in the verbal form of actions, leads to mastery of the child’s actions. Delays in verbal development lead to decreased voluntary regulation of children’s motor behavior. It is widely acknowledged that the auditory and somatosensory systems are essential in overseeing speech motor and motor acts. Multiple studies indicate that specific speech movements are orchestrated during speech to achieve motor objectives, thereby enhancing production control (Sato et al., 2013).

Prior studies have confirmed this, showing that subjects who stutter have both nonverbal sensorimotor disorders and more problems performing motor tasks (Daliri et al., 2014). Thus, levels of conscious movement control are the result of the integrated function of speech, and through this participation, the formation of the motor functional system takes place. Moreover, studies of the speech development of children with motor disorders have shown that children with motor developmental delays commonly exhibit varying degrees of speech impairment, which supports the correlation described above (Agranovich et al., 2021). Levels of brain activity in children with birth motor disorders differ from those of healthy children (Blagoveschenskiy et al., 2018).

Various factors could contribute to this outcome. For instance, an individual may have fallen behind their peers as a result of being absent from school due to illness. Children with these motor disorders may have to attend treatment, rehabilitation procedures, or surgeries throughout the year, meaning that they are absent from the educational process for long periods. In addition, their illness is likely to affect their self-esteem and social adaptation, which also has a strong impact on their overall development. It is also worth noting that the parents of these children can become so accustomed to performing everyday activities for their children that this turns into overprotection and can also affect their development.

Recent evidence has confirmed the usefulness of speech tests in predicting the risk of cognitive impairment (Sutin et al., 2019; Villalobos, 2022). Their predictive value, combined with ease of use, makes fluency tests an important screening tool for children with motor disorders in the context of rehabilitation programs.

## 6 Conclusion

This study revealed that children born with nonprogressive neuromuscular disorders (AMC or OBPP) performed poorly on speech tests compared to a control group. When conducting the semantic associative test (action-object), children with impairment in one limb demonstrated better results than those with multiple limbs impaired. This finding may provide insight into the impact of motor impairment on working, semantic, and procedural memory. Thus, verbal fluency tests have predictive value in this study’s results, making them an important screening tool for children with motor impairments in rehabilitation programs. In the context of embodied cognition theory, our results suggest that delays in motor skill development could impact cognitive functions by limiting the ability to utilize sensorimotor effects for vocabulary acquisition and conceptual understanding of objects and actions. AMC and OBPP can impact various aspects of a child’s life, affecting their education and social interactions. These children’s cognitive challenges are closely linked to their motor limitations, requiring a comprehensive approach to therapy and intervention. Understanding the complex relationship between motor impairments and cognitive functions in these children is crucial for developing comprehensive intervention strategies that support their overall development and improve their quality of life.

## Data Availability

The raw data supporting the conclusions of this article will be made available by the authors, without undue reservation.
