# Supplementary material for: Verbal fluency and semantic association deficits in children with in birth nonprogressive neuromuscular diseases
Source: Front Hum Neurosci. 2025 Feb 6;19:1499521. doi: 10.3389/fnhum.2025.1499521 (PMC11841654; doi:10.3389/fnhum.2025.1499521)
Supplement: Supplementary file 1 [file Table_1.DOCX]

| Control group | | Children with motor disorder | | | |
| --- | --- | --- | --- | --- | --- |
| N=32; μ= 9,6 | | N=36; μ=10,3 | | | |
| Male | Female | Male | | Female | |
| N=17; μ= 9,2 | N=15; μ= 10,1 | N=18; μ= 10,1 | | N=18; μ=10,2 | |
|  |  | AMC | | OBPP | |
|  |  | N=22; μ=9,2 | | N=14; μ= 10,5 | |
|  |  | Male | Female | Male | Female |
|  |  | N=12 | N=10 | N=6 | N=8 |

Supplementary Table 1. Average values by age of children with motor disorders and the control group.
